# Effect of Short-Term, Above-Optimal Temperature on the Phenology of Partially or Fully Vernalized Seedlings and Its Consequences on Grain Yield in Wheat (*Triticum aestivum* L.)

**DOI:** 10.3390/plants15192955

**Published:** 2026-09-28

**Authors:** Ildikó Karsai, Ádám D. Horváth, Borbála Illés, András Cseh, Tibor Kiss

**Affiliations:** 1 HUN-REN Centre for Agricultural Research, Agricultural Institute, H-2462 Martonvásár, Hungary; horvath.d.adam@atk.hu (Á.D.H.); illes.borbala@atk.hu (B.I.); cseh.andras@atk.hu (A.C.); 2 Food and Wine Research Institute, Eszterházy Károly Catholic University, H-3300 Eger, Hungary

**Keywords:** vernalization, transient warming, developmental plasticity, flowering time, photoperiod sensitivity, *VRN1*, *PPD-D1*, tillering

## Abstract

Due to global climate change, short periods of unusually warm temperatures occur during winter and early spring with increasing frequency. Although temperature effects on wheat development have been extensively studied during later reproductive stages, the consequences of short-term warming during early development remain poorly understood. We investigated the effect of a short-term period of warm temperature on partially or fully vernalized wheat seedlings, with particular attention to their phenological characteristics and grain-yield formation. A total of 19 wheat varieties of diverse geographic origins were studied with known allele compositions in plant developmental genes of vernalization (*VRN*) and photoperiodic response (*PPD*). The experiment consisted of six treatments in factorial combinations of three low-temperature vernalizations (30, 45 and 60 days) followed by two temperature regimes (greenhouse and two-week 25 °C plus greenhouse) under long photoperiod. The effects of transient warming were strongly dependent on the degree of vernalization fulfilment. In fully vernalized winter wheat, warming generally accelerated development across genotypes, whereas after incomplete vernalization, it frequently delayed subsequent developmental transitions, particularly in photoperiod-sensitive cultivars. Variation in responses increased with decreasing vernalization saturation, indicating that allelic differences at flowering-time loci, including *VRN* and *PPD*, contribute to the integration of temperature signals during early development. Beyond phenology, transient warming strongly influenced tiller formation and reproductive tiller number. Following complete vernalization, warming increased fertile tiller production and resulted in higher grain yield, whereas responses after partial vernalization were genotype-dependent and less favorable. Comparison with previous controlled-environment experiments further demonstrated that thermal regime, rather than mean temperature alone, strongly influences tiller survival and yield formation. Short-term warming around the completion of vernalization can have long-lasting effects on wheat development and productivity, highlighting the importance of developmental stage, genetic background and environmental context in determining crop responses to increasingly variable winter temperatures.

## 1. Introduction

Temperature is one of the most important environmental signals regulating plant growth and development throughout the life cycle. Beyond its role as an abiotic stress factor when exceeding optimal levels, temperature acts as a key developmental cue that coordinates the timing and progression of major phenological transitions [1,2,3,4,5,6]. In winter cereals, prolonged exposure to low temperatures during winter fulfils the vernalization requirement, enabling the transition from vegetative to reproductive development and contributing to winter survival [4,7,8,9,10]. Following vernalization, ambient temperature in interaction with photoperiod continues to regulate developmental processes including stem elongation, leaf and tiller production, spikelet initiation, floret development and fertilization and grain filling, ultimately influencing all major yield components [3,11,12,13,14].

The timing and duration of developmental phases are critical determinants of both ecological adaptation and grain yield in wheat [15]. During the late vegetative and early reproductive phases, the transition of the shoot apical meristem, early spike development and the onset of stem elongation determine the maximum number of spikelets and fertile florets that can be formed. Consequently, the duration of these developmental stages strongly influences the final grain number per spike, one of the principal components of grain yield [3,5,16]. The relative lengths of successive phenological phases, particularly the stem elongation period, have been shown to affect yield potential independently of the total time to anthesis [17]. Another important component of grain yield is the tillering ability and its maintenance until reaching the reproductive phase, as the number of reproductive tillers forms the basis of spike number per m^2^ [18]. Most of the tillering occurs during the vegetative growth stage, which generally ends with the onset of stem elongation. The overall extent of branching, tiller survival and fertility are affected by temperature, light, nutrient status and row spacing and are also under genetic control [18,19]. Extending developmental intervals that contribute positively to yield formation without delaying flowering has therefore emerged as a promising breeding strategy [5,17,18,20]. Achieving this objective, however, requires a detailed understanding of the environmental regulation of wheat phenology and the underlying genetic mechanisms controlling developmental timing.

In wheat, the timing of development is primarily regulated by the interaction of three main genetic pathways, each of which is composed of a series of gene cascades that regulate vernalization requirement (*VRN*), photoperiod sensitivity (*PPD*) and earliness per se (*EPS*). The *VRN-A1* gene, located on chromosome 5A, is one of the main determinants of the vernalization response, classifying wheat genotypes into winter or spring growth habits [21], while photoperiod sensitivity is influenced by the allelic composition in *PPD-D1* on chromosome 2D to the largest extent [22]. These pathways integrate environmental signals to optimize flowering time and adaptation to local environments [6,15,23,24]. While the molecular basis of vernalization and photoperiod responses has been extensively characterized, much less is known about the genetic regulation of ambient temperature responses during early reproductive development [3].

The importance of these processes has increased considerably under ongoing climate change [25,26]. Global warming is accompanied not only by a gradual increase in average temperatures but also by more frequent and less predictable weather anomalies, including short periods of unusually warm temperatures and heat waves occurring outside the traditional summer season [27,28,29,30]. Such temperature anomalies may alter plant developmental programs even when temperatures remain below levels generally considered stressful [31,32]. Based on an extrapolation of the temperature responses of 30 yield model ensembles, Asseng et al. [33] demonstrated that global warming is already reducing yield growth in most wheat-growing regions, and they predicted that global wheat production could decline by 6% with every additional °C increase in temperature. In a multi-year field experiment conducted under local growing conditions, we previously demonstrated that weather anomalies altered phenological patterns and that the extent of yield loss attributable solely to a shift in the developmental rhythm ranged from 4.5% to 20%, depending on the severity of the weather anomaly [34].

Recent climatic trends indicate that warming is not evenly distributed throughout the year. In many wheat-growing regions, the increase in temperature during late winter and early spring has exceeded the annual average [35]. This period coincides with the completion of vernalization, tillering, the vegetative-to-reproductive transition, early spike development and the initiation of rapid stem elongation. Because these developmental processes establish the potential grain number well before anthesis, warmer-than-optimal temperatures during this period may have irreversible consequences for final yield. Compared with the extensive literature on vernalization and heat stress during flowering and grain filling, relatively little attention has been paid to the biological significance of moderately elevated temperatures during the post-vernalization developmental period [14,16,26,36]. In addition, although considerable progress has been made in understanding the molecular mechanisms underlying vernalization and high-temperature stress responses [13,37], the mechanisms by which wheat senses ambient temperature and integrates these signals into developmental regulation remain poorly understood. In particular, the effects of transient periods of elevated temperature during and immediately after vernalization on plant phenology and subsequent yield formation remain poorly characterized [28]. Likewise, little information is available on the extent of genetic variation among wheat genotypes in their developmental sensitivity to elevated temperatures during these critical stages. Addressing these knowledge gaps is becoming increasingly important for wheat improvement. Detailed biological, genetic and genomic analyses of developmental regulation will facilitate the identification of flowering-time alleles that contribute to local adaptation under increasingly variable climatic conditions.

Based on the above-mentioned aspects, our objective is to improve the understanding of temperature-dependent developmental regulation during the early reproductive phase. In this experiment, we investigated the effects of a short-term period of warm ambient temperature on partially or fully vernalized wheat seedlings, with particular attention to their phenological and morphological characteristics, as well as the grain-yield-related traits. To this end, we selected 19 wheat varieties for which the allele composition of key plant developmental genes and responses to constant environmental temperature had been previously characterized by Kiss et al. [12] using a factorial combination of two constant temperature (18 °C and 25 °C) and two photoperiod levels (16H and 12H) in controlled growth chambers. In this study, we demonstrated that a two-week warm period of constant 25 °C (supra-optimal) applied at the seedling stage significantly affected both phenological and grain-yield-related traits in wheat, the direction and extent of which were substantially determined by the level of vernalization saturation, and by the allele compositions in the *VRN1* and *PPD-D1* genes. In the present study, 25 °C is referred to as an above-optimal temperature because it exceeds the temperature range generally considered optimal for vegetative development of wheat, while being substantially lower than temperatures commonly applied to induce acute heat stress [1].

## 2. Results

The same 19 wheat cultivars were earlier included in controlled growth chamber tests [12], where the effects of two constant temperatures 18 °C and 25 °C throughout the experiment on plant development were evaluated under a 16 h photoperiod regime. In that experiment, a standard low-temperature vernalization treatment of 60 days was applied. Thus, we were able to compare the phenological, morphological and grain-yield-related traits measured under those conditions with the ones from the vern60 we recorded in the present greenhouse experiments (Supplementary Table S1). In the cases of phenology and morphological traits, the correlations between the two experimental series were significant and strong. The type of associations for heading date (ZD59) are presented in Supplementary Figure S1. For this trait, the correlation coefficients were r = 0.673** between the constant 18 °C (18C) and the controlled greenhouse comparisons (GH), and r = 0.723*** between the constant 25 °C (25C) and the two-week 25C+ greenhouse comparisons (25GH), validating the results of the present experiment.

Of the three main factors (vernalization saturation, short-term warm temperature treatment and genotype), vernalization had the strongest effect on phenology, explaining between 50.5% (ZD59) and 68.2% (ZD31) of the phenotypic variance (Supplementary Table S2). The genotypic main effect was the second strongest determinant, accounting for between 8.5% (ZD31) and 18.5% (ZD59) of the variance. In contrast, the main effect of temperature was relatively small (0.4–7.9%), and temperature contributed to phenological variation mainly through its interaction with vernalization, which accounted for 17.5–21.3% of the variance.

In addition to its effects on developmental phases, the short-term 25 °C treatment applied during early plant development significantly influenced most of the morphological and grain-yield-related traits. Its effect was most pronounced on reproductive tiller number, explaining 77.0% of the trait variance. As a possible consequence, the temperature regime accounted for 45.0% of the phenotypic variance in grain yield. The interaction between temperature regime and vernalization was particularly important for average seed number (32.3%) and grain yield (20.4%), indicating that both the magnitude and direction of the effect of the two-week 25 °C treatment on these traits depended strongly on the level of vernalization saturation.

### 2.1. Plant Developmental Patterns of Wheat Seedlings as Influenced by Vernalization Saturation and Short-Term Warm Temperature

To establish the developmental profiles of the 19 wheat genotypes to the short-term warm period after various saturation levels of their vernalization requirements, principal component analysis was carried out on the data matrix of five consecutive developmental phases × two temperature regimes × 19 wheat genotypes (Figure 1). The first factor, with an eigen value of 24.7 and explaining 82.4% of the variance, stood in strong negative correlation with all the developmental phases from the six treatments. Factor 1 thus did not differentiate between the various treatments. Wheat cultivars towards the positive end of the *x*-axis developed faster, while those towards the negative end were always later, irrespective of either the vernalization length or the temperature regime. Factor 2, with an eigen value of 2.58 and explaining 8.6% of the variation, on the other hand, provided differentiation between the treatments to some extent. It demonstrated significant negative correlations with four developmental phases from ZD37 (−0.617**) to ZD59 (−0.486*) only in the v60_GH treatment and significant positive correlations with ZD37 (0.457*) and ZD39 (0.434*) only in the v30_25GH treatment. Due to the combined contribution of the two principal components, the genotypes occupied four quadrants of the PCA biplot, reflecting differences in their developmental response patterns across the treatments. The quadrants were defined by the zero coordinates of PC1 and PC2 and were therefore used as descriptive categories rather than as statistically discrete clusters. The three genotypes located in Q2 (AT12—‘Cadenza’, AT15—‘Paragon’ and AT17—‘Weebil’) were spring wheat cultivars, whereas all photoperiod-insensitive winter wheat genotypes were located in Q1 (AT1—‘Mv Toborzó’, AT5—‘Plainsmann V’, AT6—‘Mv Magma’, AT7—‘Soissons’, AT10—‘Ukrainka’). Most photoperiod-sensitive winter wheat cultivars were distributed between Q3 (AT3—‘Tommi’, AT11—‘Avalon’, AT13—‘Spark’, AT14—‘Rialto’, and AT19—‘Buster’) and Q4 (AT2—‘Mv Verbunkos’, AT4—‘Ellvis’, and AT20—‘Charger’), although two genotypes, AT8 (‘Fleischman 481’ and AT9 (‘Mv Hombár’), were positioned in Q1 together with the photoperiod-insensitive winter wheats. Genotypes in Q1, Q2 and Q3 were relatively closely grouped, whereas those in Q4 showed the greatest dispersion, primarily along PC1. The unusual response profile of AT20 (‘Charger’) under 25GH treatments largely accounted for the broad extent of the Q4 confidence ellipse and its overlap with the other quadrants.

**Figure 1:**
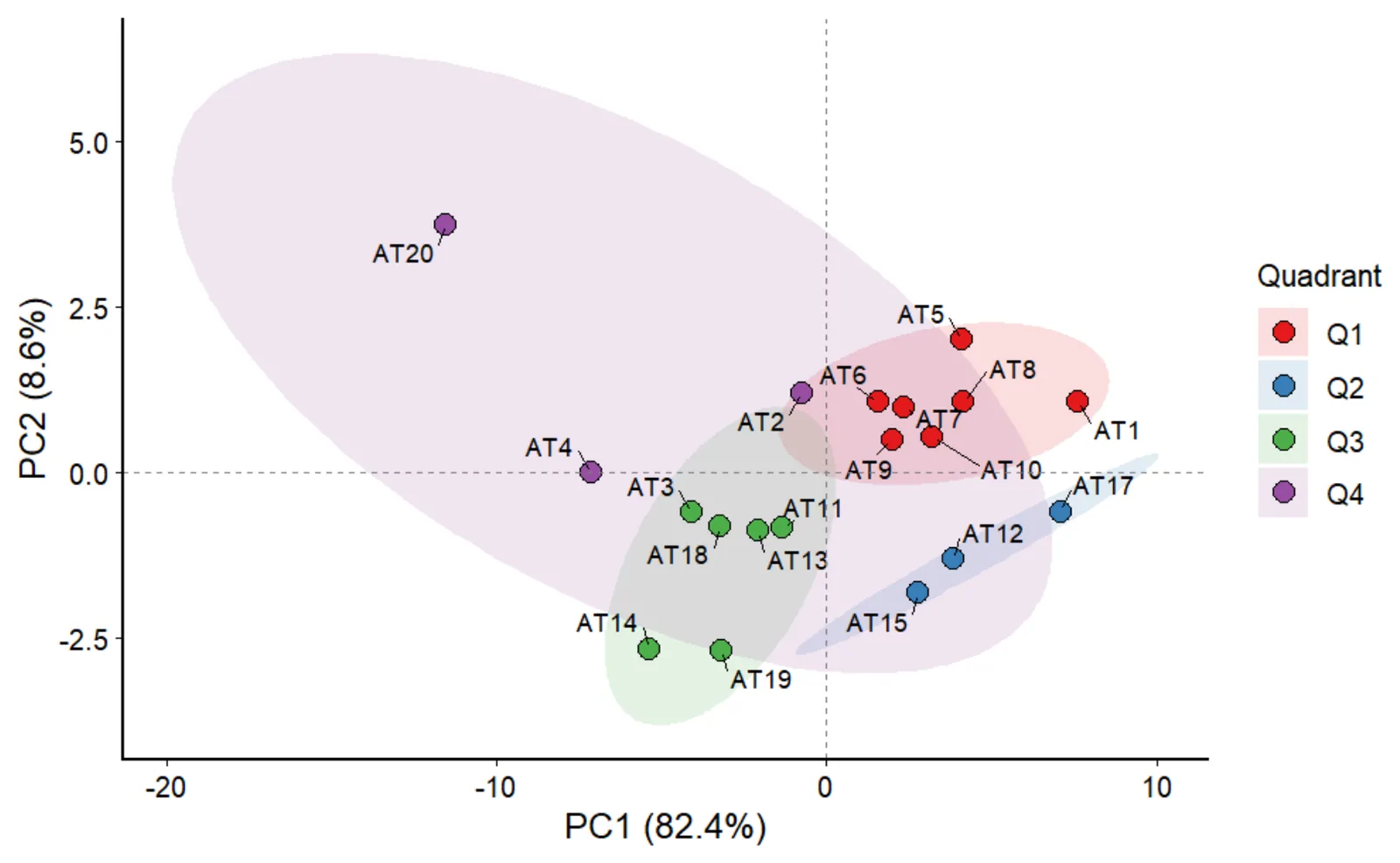
Bi-plot graph of the 19 wheat cultivars based on the principal component analysis carried out on the data matrix of 5 consecutive phenophases × 6 treatments combining the vernalization length and temperature regimes. Ellipses represent the 95% confidence regions of the respective genotype groups. (The numbers in the graph refer to the corresponding wheat genotypes as listed in Table 1. The dashed lines indicate the zero values of PC1 and PC2 used to define the four quadrants.)

**Table 1:** List of the wheat genotypes and heading dates under greenhouse conditions following different lengths of low-temperature vernalization treatments (no vernalization (v0); 15-day (v15), 30-day (v30), 45-day (v45) and 60-day long (v60) vernalization).

Abbr.	Name	Origin ^1^	Growth Habit ^2^	Photoperiod Sensitivity ^3^	Heading Date (ZD59), Days After Transplanting					
					v0	v15	v30	v45	v60	LSD_(0.05)_^4^
AT1	‘Mv Toborzó’	H	winter	Insensitive	104	75	68	60	59	2.7
AT2	‘Mv Verbunkos’	H	winter	Sensitive	126	94	78	77	73	2.9
AT3	‘Tommi’	D	winter	Sensitive	150	98	86	84	81	1.6
AT4	‘Ellvis’	D	winter	Sensitive	150	122	92	83	80	2.2
AT5	‘Plainsmann V’	USA	winter	Insensitive	112	77	68	64	59	1.7
AT6	‘Mv Magma’	H	winter	Insensitive	117	82	73	73	67	2.9
AT7	‘Soissons’	F	winter	Insensitive	120	73	71	69	66	2.7
AT8	‘Fleischman 481’	H	winter	Sensitive	98	75	69	68	63	2.8
AT9	‘Mv Hombár’	H	winter	Sensitive	112	79	71	69	68	2.8
AT10	‘Ukrainka’	UKR	winter	Insensitive	118	86	72	67	66	2.6
AT11	‘Avalon’	GB	winter	Sensitive	134	95	82	78	80	2.7
AT12	‘Cadenza’	GB	Spring ^5^	Sensitive	72	72	71	68	68	2.2
AT13	‘Spark’	GB	winter	Sensitive	97	88	83	79	74	2.0
AT14	‘Rialto’	GB	winter	Sensitive	103	96	91	85	85	2.0
AT15	‘Paragon’	GB	spring	Sensitive	77	74	73	72	71	2.6
AT17	‘Weebil’	Mex	spring	Insensitive	70	68	68	62	60	3.0
AT18	‘Savannah’	GB	winter	Sensitive	127	101	86	84	82	3.0
AT19	‘Buster’	GB	winter	Sensitive	126	91	83	83	84	2.6
AT20	‘Charger’	GB	winter	Sensitive	150	150	96	84	77	1.7

^1^ H: Hungary; D: Germany; F: France; UKR: Ukraine; GB: Great Britain; Mex: Mexico. ^2^ based on the allelic compositions in *VRN-A1*, *VRN-B1* and *VRN-D1*. ^3^ based on the allelic composition in *PPD-D1*. ^4^ least significant difference at *p* = 0.05 level. ^5^ the source of spring growth habit in cultivars: ‘Cadenza’ carries dominant spring allele at *VRN-A1* locus; ‘Paragon’ dominant spring alleles at *VRN-A1*, *VRN-B1*; ‘Weebil’ dominant allele at *VRN-D1* [12].

We compared the plant developmental response profiles characteristic to the four quarters of the bi-plot graphs in the six various treatments (Figure 2). Taking into account the ZD59 value as the final developmental stage, the spring cultivars of Q2 were always the earlier, followed by the photoperiod-insensitive winter cultivars of Q1 (including the two sensitive genotypes). There were no significant differences between these two groups in their reactions to the length of the vernalization treatment in itself; only v30 caused a slight delay in the heading of both groups compared to the similar values of v60 and v45. The difference between the two groups became apparent in how their vernalized seedlings responded to the short period of warm temperatures and in how the duration of the vernalization treatment influenced this response. In the case of the photoperiod-insensitive winter cultivars (Q1), the warm period significantly accelerated their heading following a 60-day vernalization, had no effect following 45-day vernalization, and significantly delayed heading following the 30-day vernalization. In the case of the spring cultivars, the warm period accelerated plant development in all treatments, and there were only minor differences among the three vernalization levels. The two groups of the photoperiod-sensitive winter cultivars (Q3 and Q4) were always later in all the six treatments. The length of vernalization in itself did not significantly influence the heading dates for either of the two late groups. They again differed from each other in the reaction profiles of the vernalized seedlings to the short-term warm period. In this respect, Q3 was similar to Q1 (the group of insensitive winter cultivars) in that the warm period significantly accelerated heading after v60, had no effect after v45 and caused a significant delay after v30. The heading date of the fourth quarter was the only one that the short-term warm period delayed after all the three lengths of vernalization, the extent of which increased remarkably with the decrease in the vernalization length. After the short-term warm period following v30, AT20 was not able to head (150 days), but the heading of both AT4 and AT2 also became extremely prolonged (127 and 109 days, respectively).

The intervals between the consecutive developmental phases were also significantly influenced by the two environmental factors that showed strong group specificity of the four quarters (Figure 2). In terms of which developmental interval showed the greatest variation, the insensitive winter wheat genotypes of Q1 differed most from the other three quarters. In this case, the intervals between seedling stage to the first node appearance (ZD31) and from fully emerged flag leaf to booting stage (ZD4939) were the two intervals that varied the most, with definitely opposing trends between them. While the first node appeared significantly earlier in seedlings vernalized for 60 and 45 days when treated with short-term 25 °C temperature, after a 30-day vernalization, the same warm temperature led to a much prolonged first node appearance. This change in ZD31, however, was more or less counteracted with the changes in the interval between the fully emerged flag leaf and booting stage that were especially prolonged after v60 and v45 due to the warm temperature that completely disappeared after v30. In the cases of the other three quarters, instead of the interval to first node appearance, the interval between first node and the flag leaf appearances (ZD3731) proved to be much more variable in addition to the interval of fully emerged flag leaf and booting (ZD4939). The type and extent of changes in ZD3731 due to the vernalization and warm temperature were quarter-specific. For the photoperiod-insensitive genotypes (Q1), this interval was more or less stable across the six treatments. For the spring genotypes (Q2), the two-week warm temperature decreased this interval length irrespective of the vernalization treatments, while in the case of the late genotypes in Q4, ZD3731 was lengthened due to the warm treatment, the ratio of which significantly increased with the shortening of the vernalization treatment. For the photoperiod-sensitive winter wheats in Q3, the direction of the warm-period-induced changes in the ZD3731 interval depended on the length of vernalization. It shortened ZD3731 to the largest extent after 60-day vernalization, to a smaller extent after 45-day vernalization, while it significantly increased this interval length after 30-day vernalization.

### 2.2. Effect of Vernalization Saturation and Short-Term Warm Temperature at Seedling Stage on Morphological and Yield-Related Traits

In addition to plant development, the length of vernalization and the short-term warm period significantly influenced several morphological and grain-yield-related traits as well (Supplementary Table S1). To demonstrate these changes, we chose the two extremes, the fully (v60) and partially vernalized (v30) treatments (Figure 3), comparing the averages of the 19 wheat cultivars and the four groups corresponding to the four quarters of the bi-plot graph (Figure 1). Concerning the leaf characteristics of the main stem, the short-term warm period resulted in decreased phyllochron, and a slight increase in the final leaf number when it occurred after v60—especially. in the photoperiod-insensitive genotypes (Q1), while the sensitive genotypes of Q3 showed no significant change in either parameter. When the warm period occurred after a partial vernalization (v30), the leaf appearance rate became significantly slower, which was also accompanied with a significant increase in the final leaf number. These tendencies were the strongest for the Q4 genotypes, while being less marked for the spring growth habit group (Q2). For the plant dimensional values, the last internode length was the most sensitive to the short-term warm period, mostly independently from the vernalization saturation level; this parameter decreased in most of the groups.

The reproductive tiller number of the grain-yield-related traits was most strongly influenced by the short-term warm period, especially in v60. In this case, there was a 191% increase averaged over the 19 cultivars with a range between 161% (Q4) and 233% (Q3) of the four quarter groups. To a smaller extent, this increasing effect of the short-term warm period on RT could also be detected after a partial vernalization (v30), with the interval between 66% (Q4) and 162% (Q1). In the group of photoperiod-insensitive cultivars, the warm-temperature-induced RT increase was relatively stable across the vernalization treatments. At v60, the warm-period-induced increase in RT was accompanied with a relatively stable average grain number and weight resulting in a significantly increased grain yield (153% averaged over the 19 wheat cultivars), as well. In the case of v30, however, the average grain weight per spike decreased in most of the groups, leading to a reduced or a control-level grain yield at most. It is interesting to note, that in v60, the group of photoperiod-sensitive cultivars (Q3) responded most positively to the short-term warm period with respect to their grain-yield increase. This was due not only to their highest increase in RT, but also to a significant increase in the average grain weight per spike.

We examined how the interval lengths between the various consecutive developmental phases influenced the grain-yield-related traits. The associations proved to be strongly dependent on the vernalization saturation level and on the short-term warm period as demonstrated by the path analyses of the two extreme vernalization treatments of v60 and v30 (Figure 4, Supplementary Figure S2). Under the fully vernalized conditions followed by controlled greenhouse plant raising (v60_GH), only two developmental interval lengths showed weak negative associations with any of the grain-yield-related traits. The length of the flag leaf emergence (ZD3937) negatively affected the main-ear thousand-kernel weight. The length between the booting and fully emerged flag leaf (ZD4939) negatively influenced the grain weights in the main and side ears and the thousand-kernel weight via the plant grain yield as well. The two-week 25 °C treatment of v60_25GH magnified the significance of the various developmental intervals in contributing to grain-yield formation. Thus, the negative effect of the timing of the first node appearance on the thousand-kernel weight became most apparent, while the negative associations between booting and heading (ZD5949) on the average seed number and weight and thus on grain yield intensified. Under these conditions, however, the length of flag leaf development (ZD3731 and ZD3937) was positively associated with the grain numbers both in the main and side ears, reflected in the grain yield as well. Under the partially vernalized conditions (v30), the overwhelming negative effect of the timing of first node appearance on the thousand-kernel weight was apparent in both ambient temperature regimes. The role of the ZD3731 interval (between flag leaf and first node appearance) also intensified. Under the control condition (GH), it negatively influenced the reproductive tiller number, but following the two-week 25 °C treatment, this association was further intensified and was coupled with a strong reduction in the grain weights as well. As a result of these effects under the v30_25GH condition, the ZD3731 interval length was observed to be one of the most decisive factors in grain-yield formation.

## 3. Discussion

Although numerous studies have investigated the effects of elevated ambient temperature during the later reproductive stages of wheat development, particularly from the onset of stem elongation onwards, comparatively little is known about how a transient warm period affects seedlings during the early developmental phases around the completion of vernalization [3,14]. By assessing the responses of 19 wheat cultivars to a short-term warm treatment applied at the seedling stage, we demonstrated that this early environmental stimulus was sufficient to induce significant changes not only in phenological development throughout the life cycle but also in grain-yield-related traits. The magnitude and direction of these responses depended strongly on the degree of vernalization saturation and on the allelic composition of the *VRN* and *PPD* loci. The influence of the latter became increasingly pronounced as the rate of vernalization decreased, with the exception of the three spring wheat cultivars.

In winter wheat, the short-term warm treatment significantly accelerated plant development when the vernalization requirement had been fully satisfied, largely irrespective of the *PPD-D1* allele. However, the importance of *PPD-D1* increased as vernalization became less complete. Photoperiod-insensitive genotypes exhibited remarkably similar responses to the treatments, clustering closely in Quarter 1 of the biplot, whereas photoperiod-sensitive cultivars displayed substantially greater variation, being distributed across three quadrants, with a few clustering together with the insensitive genotypes, contrary to the findings of Kiss et al. [12]. The broad range of responses among photoperiod-sensitive cultivars indicates that variation at *PPD-D1* alone cannot fully explain the observed phenotypic diversity, suggesting that additional genetic factors contribute to the perception and integration of transient warm episodes during early wheat development [12,38]. Besides the known variation at the major *VRN* and *PPD* loci, additional components of the flowering-time network, including earliness-per-se loci and downstream flowering regulators, may contribute to this genotypic diversity. However, identifying their individual contributions requires genetic analyses in larger and genetically structured populations and cannot be resolved from the present set of 19 cultivars.

The effect of temperature on reproductive development is strongly dependent on day length [8,12]. In general, previous studies have shown that elevated temperatures accelerate reproductive development under long-day conditions but delay the early stages of reproductive development under short-day conditions [1,3,12,14,39,40]. In the present study, the short-term warm treatment accelerated plant development relative to the greenhouse control only when the vernalization requirement had been mostly satisfied. By contrast, in our previous controlled-environment experiment, the same 19 cultivars developed considerably faster throughout the later developmental stages under a constant temperature of 18 °C and the same long-day photoperiod following an identical vernalization treatment (Supplementary Table S1; Kiss et al. [12]).

Furthermore, the effects of constant and diurnally fluctuating temperatures differ significantly, and these responses are further influenced by the magnitude of the temperature fluctuations as well as by whether the night-time temperatures are lower or higher than the daytime temperatures [9,14,41,42,43]. It should be noted that during the first two weeks of the plant growing conditions, 25GH differed from GH not only in the mean temperature but also in the diurnal thermal amplitude, since 25GH was maintained at a constant 25 °C whereas GH temperatures fluctuated diurnally. The individual contributions of these two thermal components therefore cannot be separated. However, this difference was restricted to the two-week treatment period, after which both groups developed under the same greenhouse conditions. Furthermore, the consistency of genotype-dependent developmental patterns across independent experiments conducted under different thermal regimes (Supplementary Table S1) indicates that the major genotypic responses were not specific to the particular GH–25GH temperature contrast. In addition, applying the warm treatment after partial (30-day) vernalization significantly delayed all subsequent phenological stages in every winter wheat genotype, even under long-day conditions. This delay was most pronounced in several photoperiod-sensitive cultivars. These findings highlight the complexity of the interactions between photoperiod and ambient temperature in regulating phenological development. These interactions are further modified by the degree of vernalization saturation, the duration and amplitude of temperature fluctuations, the genetic architecture of the genotype and the developmental stage of the plant at the time of exposure, among other factors [8,40,44].

It is also important to note that the response of partially vernalized wheat plants to a short-term warm period is likely to be biologically relevant under natural conditions, as it may prevent precocious reproductive development during transient warm spells in winter [4,32]. Our results further suggest that, under the long-day conditions used here, the response of juvenile, partially vernalized plants to transient warming was largely independent of photoperiod. The persistence of these effects throughout later developmental stages suggests that transient thermal events occurring around the completion of vernalization may alter the developmental state of wheat plants in a way that influences subsequent growth transitions. Although the molecular basis of this response remains unknown, it may involve interactions with the vernalization memory system, in which stable regulation of *VRN* genes maintains competence for reproductive development after environmental induction [45,46].

Our results are consistent with previous reports showing that the durations of individual phenological phases exhibit greater plasticity than the heading date itself, and that variation in these developmental intervals is often more strongly associated with grain-yield-related traits than the heading date alone [12,47,48]. Ambient temperature again had significant effects on the interval length that depended on the temperature level applied. There were clear differences between these processes under the two temperature regimes. In particular, the characteristics of intensive stem elongation showed definite changes under 25 °C, the type of which was genotype-dependent, mostly connected to *PPD-D1* [12]. Although these responses are genotype-dependent, the significant treatment and genotype × treatment interaction effects complicate the identification of robust relationships between developmental intervals and yield-related traits [44]. Consequently, the confidence with which these associations can be translated into practical breeding strategies for improving ecological resilience remains limited [5,49,50]. To establish reliable and broadly applicable relationships, large-scale experiments using the same genetic materials across diverse environmental conditions are required. Such datasets would provide the scale and diversity necessary for the effective application of deep-learning approaches to refine crop growth models and improve their predictive performance [26,40,51,52].

Although the heading dates of wheat genotypes are generally well correlated between field and controlled-environment experiments (Supplementary Figure S3a), early vegetative development differs markedly between these conditions [34,48]. Under field conditions following autumn sowing, leaf emergence, tiller initiation and vegetative apex development begin well before vernalization is fully satisfied, typically under relatively mild temperatures combined with short and progressively decreasing day lengths. Whenever temperatures remain above freezing, these developmental processes continue throughout winter, resulting in plants with several leaves and tillers by late winter or early spring, when the transition of the shoot apices to the reproductive phase also occurs (Fowler et al. [4]; Supplementary Figure S3b). Several field studies have shown that transient warming during late winter or early spring advances spring regrowth, promotes tillering and spike development, and can ultimately increase grain yield when warming occurs within an appropriate developmental window [16,36,53]. Plants grown under controlled conditions, however, usually complete vernalization at the one- or two-leaf stage, without tillers and with the shoot apex remaining vegetative despite the vernalization requirement being fully satisfied. Consequently, the period available for tiller initiation is considerably shorter under controlled conditions [3]. Nevertheless, our results demonstrate that a transient warm period applied immediately after vernalization can elicit responses similar in direction to those observed under field conditions.

A particularly interesting outcome of the present study was the effect of the short-term warm treatment on tillering and the number of fertile tillers under greenhouse conditions compared with our previous controlled-environment experiment conducted at constant temperatures of 18 and 25 °C [12]. These comparisons highlight the importance not only of the mean temperature but also of the thermal regime in determining tiller development. Regardless of temperature level, plants grown under constant temperatures produced significantly more tillers than those grown under the naturally fluctuating temperatures of the greenhouse (Supplementary Table S1). However, a substantially larger proportion of these tillers aborted before heading, whereas under greenhouse conditions, more than 70% developed into fertile ears. As a consequence of this improved tiller survival, the transient warm treatment applied at the seedling stage produced the greatest number of fertile tillers and, ultimately, the highest grain yield among the four experimental environments. Together with previous field studies demonstrating enhanced tillering following transient warming during late winter or early spring, this suggests that, once vernalization has been completed, the beneficial effects of transient warming may be largely independent of photoperiod.

The exact physiological and genetic mechanisms underlying the contrasting effects of transient warming after shorter and prolonged vernalization, however, cannot be determined from the present experiment, as vernalization progress was characterized phenologically and no physiological, biochemical or molecular markers were measured. Elucidation of these mechanisms will therefore require further targeted studies combining detailed phenology with physiological and molecular characterization of vernalization status.

## 4. Materials and Methods

### 4.1. Plant Materials

To examine the effects of a short-term warm period after vernalization on plant development and yield formation, 19 wheat cultivars of diverse geographic origins were included in the experiments. Table 1 contains information on the origin, growth habit and photoperiod sensitivity of the 19 cultivars. The latter two features are based on the determination of the actual allelic compositions in the *VRN1* and *PPD1* loci [12]. The ambient temperature responses of these genotypes were previously determined in association with photoperiod in controlled growth chambers, the results of which have already been published by Kiss et al. [12]. Prior to the present experiment, the vernalization responses of the genotypes were also determined under greenhouse conditions with the application of 0, 15, 30, 45 or 60 days of low-temperature treatments (v0, v15, v30, v45, v60, respectively), using the same methodology as described below. Following the five different vernalization treatments, the heading dates of the genotypes were measured in days, where the number of 150 stands for the category of not heading out (Table 1). After 30-, 45- and 60-day vernalization treatments, all 19 genotypes reached heading. Thus, v30 represented the minimum vernalization duration sufficient to enable heading in all genotypes, whereas v60 represented a substantially more advanced degree of vernalization fulfilment. The response of heading date to increasing vernalization duration was used as a phenological indicator of vernalization fulfilment, with a plateau beyond which no further significant decrease in heading time occurred being considered indicative of saturation of the vernalization requirement. Based on the data presented in Table 1, such a plateau could not be unequivocally established for every genotype; nevertheless, heading time reached its lowest value following v60. Therefore, for simplicity and consistency throughout the manuscript, v30 and v60 are hereafter referred to operationally as partially and fully vernalized, respectively.

### 4.2. Phenotypic Examinations

The experiment was conducted in the greenhouse and Phytotron facilities of HUN-REN ATK (Martonvásár, Hungary). It consisted of six treatments in factorial combinations of three vernalization levels, and two ambient temperature regimes. For all the treatments, the germinated seedlings were vernalized for 30 (v30), 45 (v45) or 60 (v60) days in peat blocks (Jiffy^®^, Zwijndrecht, The Netherlands) at 4 °C, and short days (9 h light period in a 24 h day). During vernalization, a low light intensity (photosynthetic photon flux density (PPFD) of 20 μmol m^−2^ s^−1^) was applied according to the standard vernalization protocol routinely used at our institute for both experimental and breeding purposes [12]. This relatively low light intensity is used to maintain viable seedlings while limiting vegetative growth during prolonged exposure to low temperature. The vernalized plantlets of one to two leaf stages were then transferred to pots containing a 3:2:1 mixture of about 1.5 kg of garden soil, compost and sand. For each genotype × treatment combination, four plants were grown in two replicate pots, with two plants per pot, corresponding to an average plant density of approximately 60 plants m^−2^. Phenological and plant-level traits were recorded separately for each individual plant, and the four individual observations were used as replicates in the statistical analyses [12]. The three vernalization treatments were so scheduled that the plants were transplanted into pots at the same time. Half of the pots were placed directly in the greenhouse as controls, while the other half were transferred to a CONVIRON growth chamber (PGV-36, Conviron, Winnipeg, MB, Canada) where they remained for two weeks and were then moved back to the greenhouse. In the greenhouse (GH), the natural light conditions of late winter−early spring were supplemented with additional lighting by metal halide lamps at a light intensity of 170 μmolm^−2^s^−1^ (PPFD) to ensure the flowering inductive long-day conditions of a 16 h photoperiod. The temperature was kept in the range of 15–21 °C throughout the experiment. The short-term treatment of supra-optimal temperature after vernalization (25GH) was carried out in a controlled growth chamber, where a 16 h photoperiod was provided with a light intensity of 170 μmolm^−2^s^−1^ (PPFD) at the plant level by wide-spectral metal halide lamps, and a temperature level of 25 °C ± 1 °C was kept constant day and night for two weeks. Thus, in the first two weeks, there were differences between the greenhouse and the transient elevated temperature treatments not only in the temperature level but also in the lack or presence of a diurnal temperature profile. The six treatments were 60-day vernalization followed by greenhouse (v60_GH) or short-term high temperature and greenhouse conditions (v60_25GH), 45-day vernalization followed by greenhouse (v45_GH) or short-term high temperature and greenhouse conditions (v45_25GH), and 30-day vernalization followed by greenhouse (v30_GH) or short-term high temperature and greenhouse conditions (v30_25GH).

From the start of the experiment, the plants were measured at 5-day intervals to determine their decimal leaf stage [54], making it possible to calculate the phyllochron (speed of leafing out, i.e., number of days required for fully developing one extra leaf on the main stem). The phenophases were defined by Zadoks’ scale [55] as follows: appearance of the first main stem node (ZD31), appearance and full expansion of the flag leaf (ZD37 and ZD39), booting stage (ZD49) and heading (ZD59). In addition, final leaf number (FLN), final plant height (FPH), last internode length (LIN) and spike length (SL) were also determined on the main stem. The experiment was terminated after 150 days. The plants were grown to full maturity and the following yield components were evaluated on each plant: number of reproductive tillers (RT), number and weight of grains and thousand-kernel weight on the main stem (MSN, MSW and MTKW, respectively), total and average number and weight of grains and thousand-kernel weight on the side tillers (SSN, ASN, SSW, ASW, and ATKW, respectively), and total grain yield per plant (GY).

### 4.3. Statistical Analyses

Basic statistical analyses were performed using ANOVA, REML or regression procedures, as appropriate, in Genstat Twenty-fourth Edition (VSN International Ltd., Hemel Hempstead, UK). For the present factorial experiment, Genstat identified the design as orthogonal, and ANOVA was performed according to the vernalization × temperature × genotype factorial structure, with replication included in the model. The phenotypic diversity of the 19 wheat cultivars based on the trait matrix (19 genotypes × 6 environments × 16 phenological traits) was established using hierarchical clustering based on the UPGMA grouping module in Statistica 6 (StatSoft, Inc., Tulsa, OK, USA). In four different environments, the networks between various developmental interval lengths and grain-yield-related traits were visualized with R qgraph 1.9.2 [56]. The correlation heatmaps were visualized by the metan 1.1.0 R package [57]. Principal component analysis (PCA) was performed in R version 4.5.0 [58]. The PCA results were visualized using ggplot2 4.0.2 R package [59], with genotype labels positioned using ggrepel 0.9.6 R package [60]. Comparisons among the four PCA-derived groups were performed separately for each trait and treatment using one-way ANOVA followed by Tukey’s honestly significant difference (HSD) test. Statistical significance was considered at *p* < 0.05, and compact letter displays were generated using multcompView 0.1.12 R package.

## 5. Conclusions

The present study demonstrates that a short-term warm period during the seedling stage can induce long-lasting changes in wheat development, extending beyond phenology to influence tillering, reproductive tiller formation and, ultimately, grain yield. The magnitude and direction of these responses depended primarily on the degree of vernalization saturation and were further modulated by allelic variation at the major flowering-time loci, *VRN* and *PPD*. In fully vernalized winter wheats, transient warming generally accelerated development irrespective of photoperiod sensitivity, whereas under incomplete vernalization, considerably greater genotypic variation was observed, indicating the involvement of additional genetic factors. Our findings, together with previous field observations, indicate that similar developmental responses may occur following transient warming under both controlled and field conditions. Once vernalization has been completed, the beneficial effects of short-term warming appear to depend less on photoperiod and more strongly on the developmental status of the plant. These results provide new insight into the integration of temperature, vernalization and photoperiod signals during early wheat development and improve our understanding of how temporary warm periods during winter may influence crop performance. They also establish a physiological framework for future genetic analyses aimed at identifying the loci controlling developmental plasticity and adaptation to increasingly variable winter climates.

## Figures and Tables

**Figure 2:**
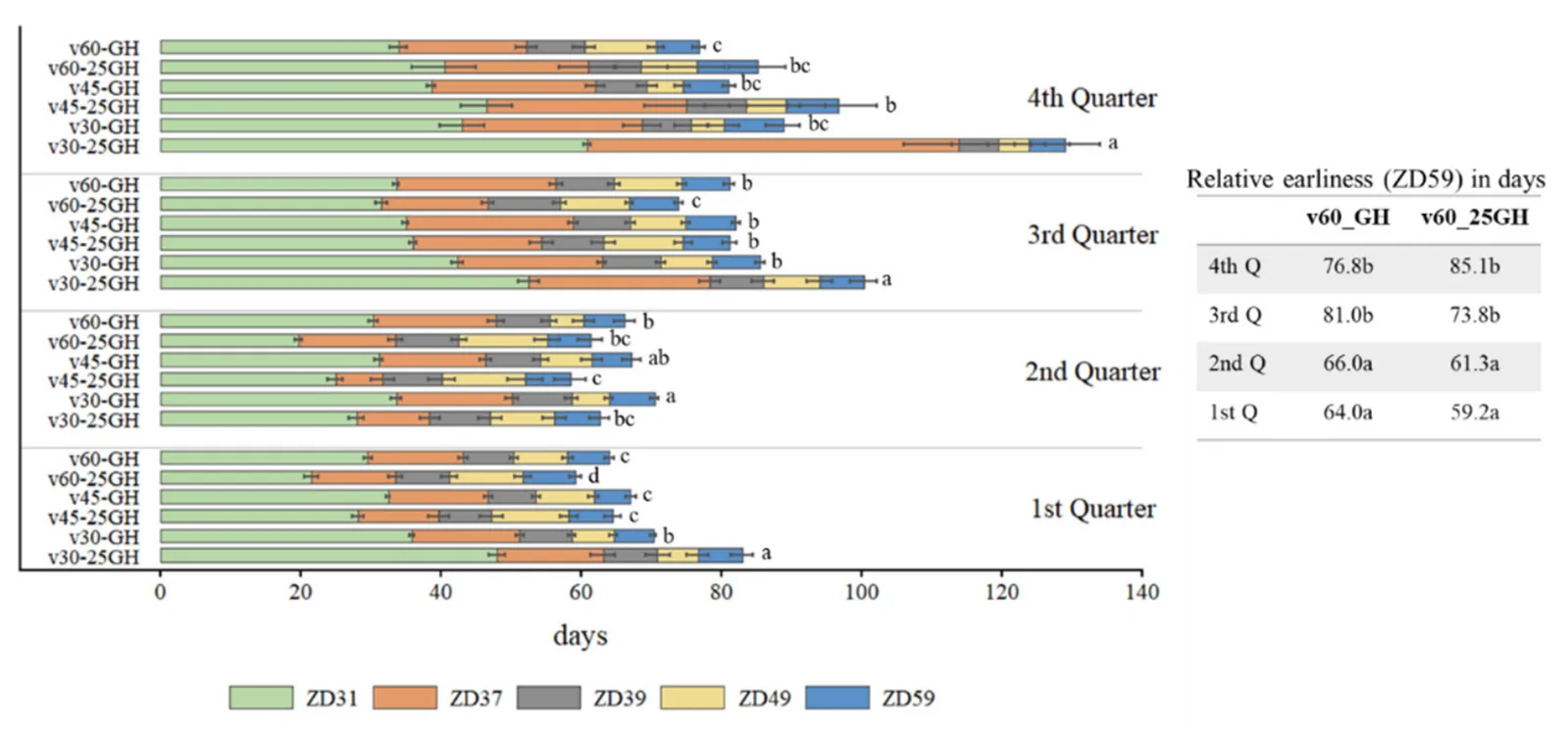
Plant developmental patterns of the wheat genotype groups of the four quarters of the bi-plot graph from first node appearance (ZD31) to full heading (ZD59) under the six treatments of three vernalization lengths and two temperature regimes, including characterization of the relative earliness of each quarter represented by the heading date values (ZD59) after full vernalization (v60). Different letters indicate significant differences in ZD59 among the six treatments within each quadrant, based on paired Student’s *t*-tests. Treatments sharing at least one letter are not significantly different.

**Figure 3:**
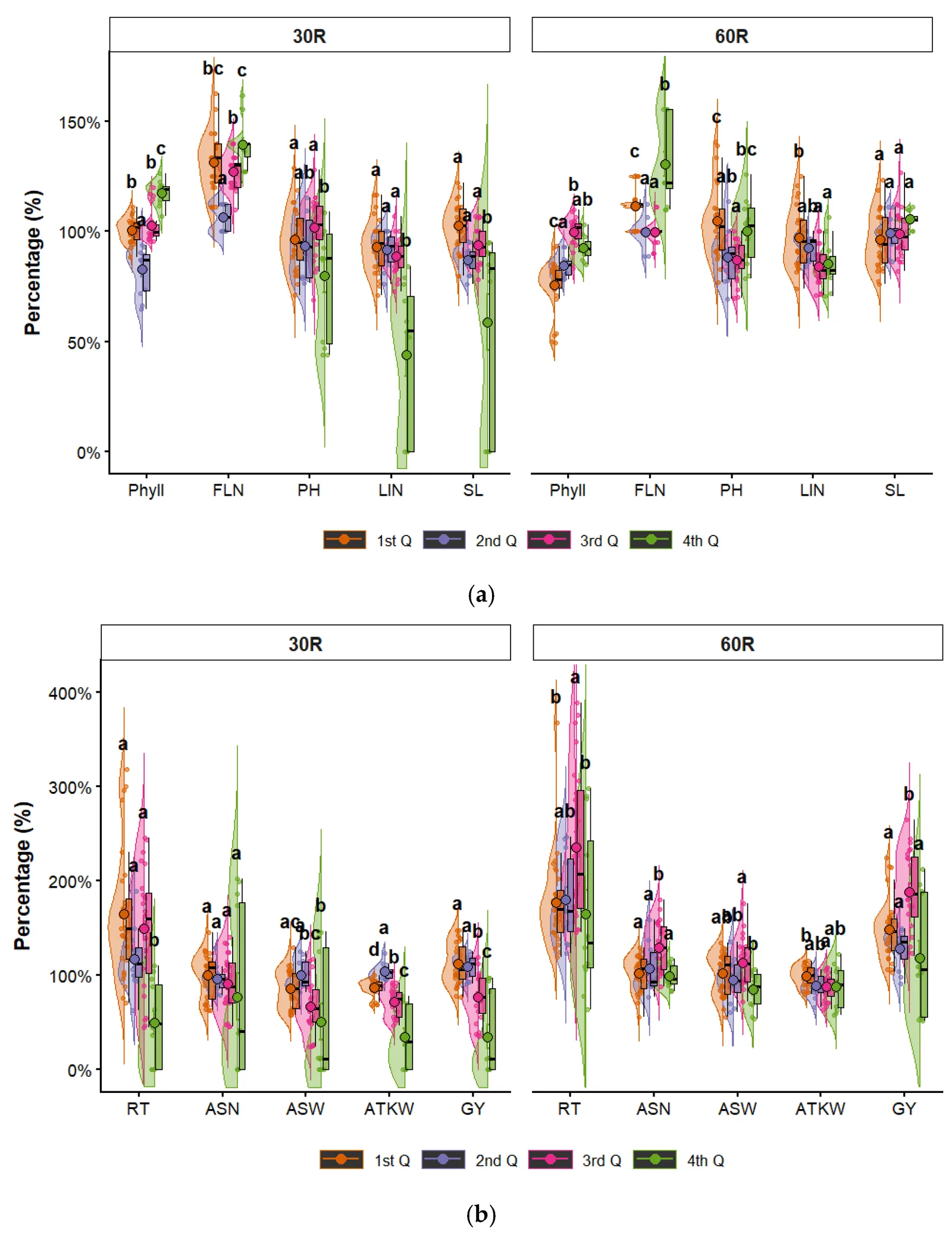
Effects of the two-week 25 °C treatment of the wheat seedlings vernalized for 60 or 30 days on (**a**) morphological and on (**b**) grain-yield-related traits for the 19 wheat cultivars and for the 4 groups of the bi-plot graph quarters, as expressed in percentages of the corresponding control greenhouse values. (60R or 30R—percentages in control following 60- or 30-day vernalizations). Half-violin plots represent the distribution and density of individual observations within each group, while boxplots indicate the median and interquartile range. Points represent individual observations, and bars represent group means with error bars indicating the standard error (SE). Horizontal lines within the boxes indicate the group median. Letters indicate statistically significant differences among the four groups within each trait and treatment according to one-way ANOVA followed by Tukey’s HSD test (*p* < 0.05). Trait abbreviations are the following: (**a**) Phyll—phyllocron, FLN—final leaf number, PH—final plant height, LIN—last internode length, SL—spike length; (**b**) RT—reproductive tiller number, ASN—average seed number per spike, ASW—average seed weight per spike, ATKW—average thousand-kernel weight, GY—plant grain yield.

**Figure 4:**
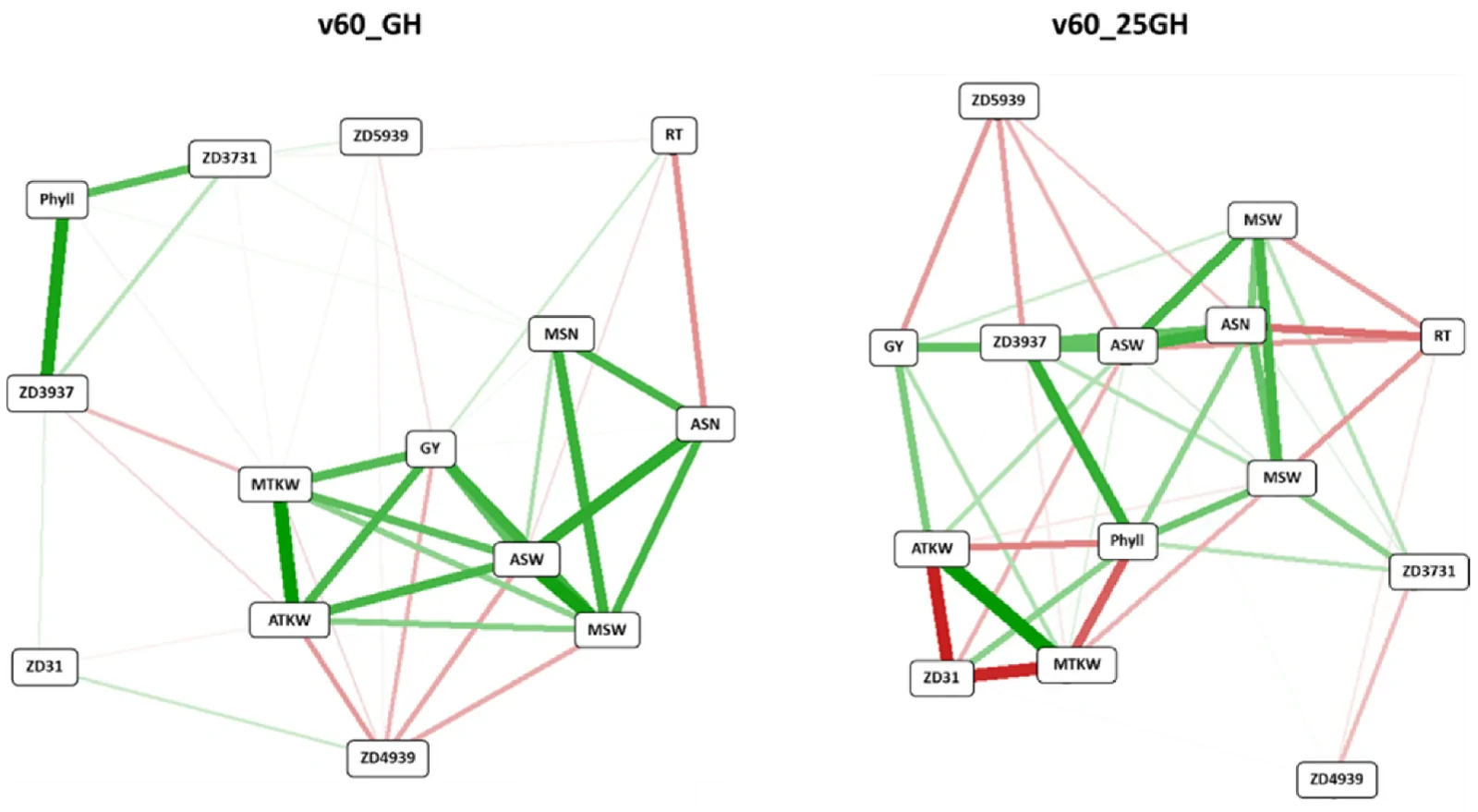

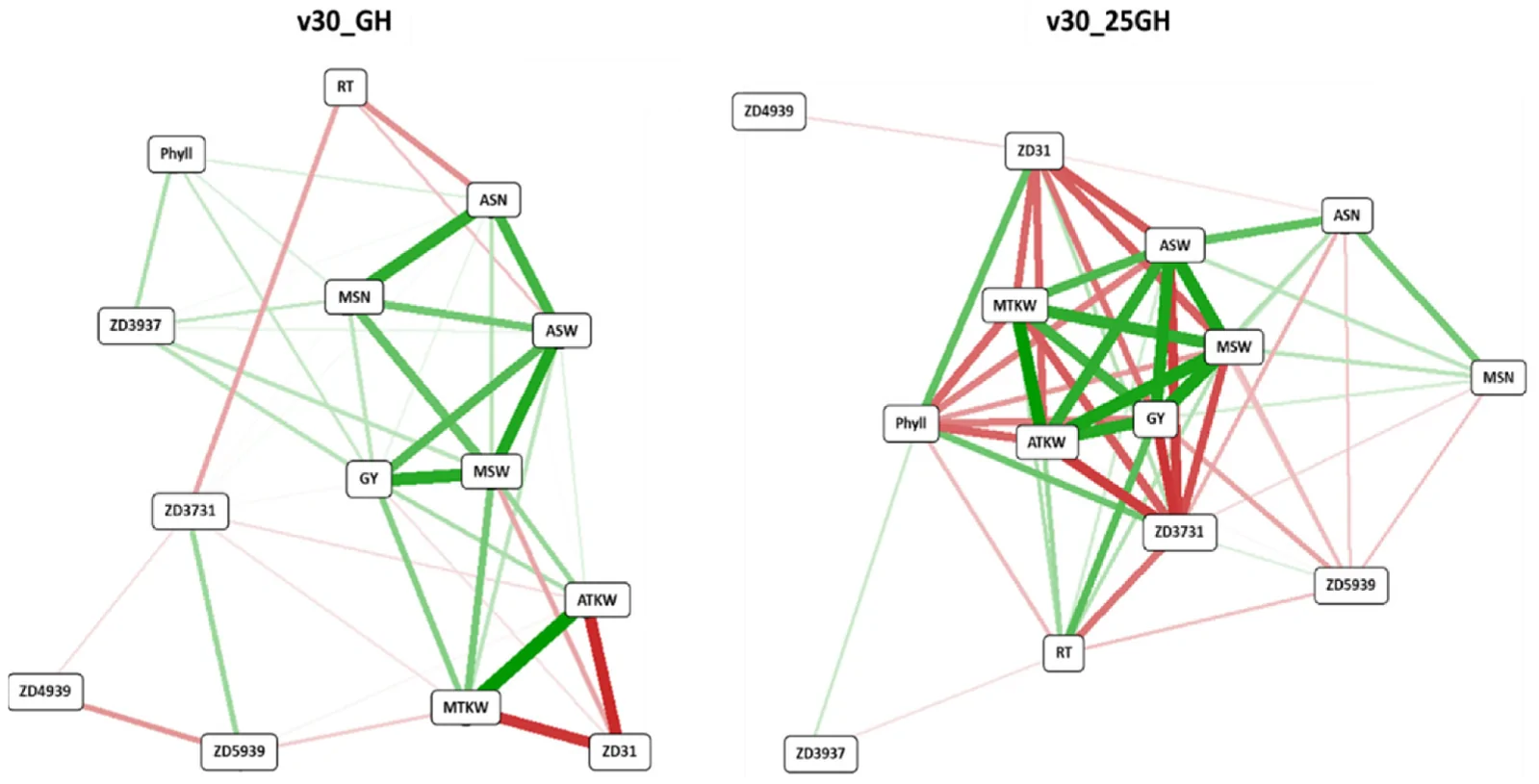
Association networks between the various developmental interval lengths and grain-yield-related traits in the group of 19 wheat cultivars under four environments, combining two vernalization lengths (60- or 30-day) with two ambient temperature regimes (control greenhouse (GH) or two-week 25 °C followed by greenhouse conditions (25GH)). Green lines indicate positive correlations, whereas red lines indicate negative correlations. Line thickness represents the strength of the correlation. Trait abbreviations: ZD31—appearance of the first main stem node, ZD3731—the interval between appearance of the first main stem node and appearance of the flag leaf, ZD3937—the interval between appearance of the flag leaf and full expansion of the flag leaf, ZD4939—the interval between full expansion of the flag leaf and booting, ZD5949—the interval between booting and heading, Phyll—phyllocron, RT—reproductive tiller number, MSN—number of grains on the main stem, MSW—weight of grains on the main stem, MTKW—thousand-kernel weight on the main stem, ASN—average seed number per spike, ASW—average seed weight per spike, ATKW—average thousand-kernel weight, GY—plant grain yield.

## Data Availability

The original contributions presented in this study are included in the article/Supplementary Materials. Further inquiries can be directed to the corresponding authors.

## Supplementary Materials

The following supporting information can be downloaded at: https://www.mdpi.com/article/10.3390/plants15192955/s1.
