# Supplementary material for: Effect of Short-Term, Above-Optimal Temperature on the Phenology of Partially or Fully Vernalized Seedlings and Its Consequences on Grain Yield in Wheat (Triticum aestivum L.)
Source: Plants (Basel). 2026 Sep 28;15(19):2955. doi: 10.3390/plants15192955 (PMC13666847; doi:10.3390/plants15192955)

Supplementary Table S1. Comparisons between the databases of developmental, morphological and grain yield related traits achieved in two independent experiments for the same 19 wheat cultivars after 60-day vernalization treatment; sets at the two constant temperatures of 18 °C and 25 °C originate from a controlled growth chamber experiment, which was already published in Kiss et al. [12]; while the two sets of GH and 25GH are from the present experiment carried out under greenhouse conditions.

Traits	Average values of 19 wheat cultivars at[1]				Correlation coefficients (r) between two environments[2]				
	18C	25C	GH	25GH	18C-25C	GH-25GH	18C-GH	25C-25GH	18C-25GH
ZD31 (days)	37.9a	43.3a	31.7b	27.3c	0.97***	0.76***	0.71***	0.88***	0.91***
ZD37 (days)	42.7b	54.6a	49.5ab	42.0b	0.87***	0.67***	0.50*	0.76***	0.92***
ZD39 (days)	47.7c	58.7ab	57.3a	50.7b	0.87***	0.75***	0.54*	0.74***	0.90***
ZD49 (days)	52.0c	61.7ab	65.6a	60.9b	0.87***	0.78***	0.64**	0.72***	0.90***
ZD59 (days)	59.1c	65.8ab	71.7a	68.2b	0.87***	0.75***	0.67**	0.80***	0.93***
Phyll (days)	5.4c	4.9d	8.2a	7.1b	0.82***	0.69***	0.61**	0.62**	0.69***
FLN (No.)	10.8b	12.0a	8.8d	9.5c	0.93***	0.38	0.46*	0.82***	0.92***
FPH (cm)	36.6c	28.2d	46.9a	44.4b	0.70***	0.94***	0.91***	0.76***	0.82***
LIN (cm)	17.3c	12.7d	23.7a	21.b	0.52*	0.93***	0.90***	0.63**	0.86***
SL (cm)	9.1a	6.2b	9.4a	9.2a	0.22	0.79***	0.56*	-0.56*	0.33
MSN (No.)	53.2a	24.8c	46.5b	49.4ab	0.15	0.61**	0.44	-0.28	0.65**
MSW (g)	2.09a	0.54b	2.09a	1.91a	0.39	0.64**	0.39	0.22	0.60**
MTKW (g)	39.4b	19.2c	45.0a	39.8b	0.58**	0.70***	0.61**	0.47*	0.74***
TLM (No.)	7.8a	5.5b	2.2d	3.8c	0.81***	0.27	0.69**	0.31	0.24
RT (No.)	2.0b	1.4c	1.6c	2.9a	0.71***	0.49*	0.20	0.44	0.61**
ASN (No.)	41.9a	18.9c	37.5b	41.2a	0.20	0.35	0.15	0.41	0.75***
ASW (g)	1.57a	0.41b	1.53a	1.53a	0.53*	0.63**	0.30	0.71***	0.78***
ATKW (g)	38.1b	19.1c	40.7a	37.3b	0.70***	0.67**	0.55**	0.58**	0.84***
GY (g)	4.6b	1.1d	4.0c	5.8a	0.45*	0.29	0.01	0.54*	0.54*

[1] the values followed by the same letter are not significantly different from each other based on the Student T-test

[2] r values are significant at *0.05, **0.01 or ***0.001 levels, respectively

Trait abbreviations:

ZD31 – first node appearance
ZD37 – flag leaf emergence
ZD39 – flag leaf fully expanded
ZD49 – booting stage, ears in the flag leaf sheath
ZD59 – heading, the ears fully emerged from the flag leaf sheath
Phyll – speed of leafing out
FLN – final leaf number on the main stem
FPH – final plant height of the main stem from soil surface to the bottom of the ear
LIN – last internode length on the main stem
SL – spike length on the main stem
MSN – grain number in the main ear
MSW – grain weight in the main ear
MTKW – thousand kernel weights in the main ear
TLM – maximum tiller number
RT – number of reproductive tillers
ASN – average grain number per spike
ASW – average grain weight per spike
ATKW – average thousand kernel weight
GY – total grain yield per plant

Supplementary Table S2. Variance components of the three factors – three vernalization lengths (V), two temperature regimes (T) and 19 wheat genotyped (G) – on different developmental, morphological and grain yield related traits. *indicates a significance level of P < 0.05, ** indicates a significance level of P < 0.01, while *** indicates a significance level of P < 0.001.

	**Variance components (σ²) of factors as expressed in %**							
**d.f.**	**Vern (V) 2**	**Temp (T) 1**	**Geno (G) 18**	**V x T 2**	**V x G 36**	**T x G 18**	**V x T x G 36**	**Rep 3**
**Traits[1]**								
ZD31	68.2***	2.1***	8.5***	17.5***	0.6***	2.1***	0.9***	0.0
ZD37	58.5***	0.4***	14.4***	21.3***	1.0***	3.9***	0.5***	0.0
ZD39	58.6***	0.9***	15.3***	20.1***	0.9***	3.8***	0.5***	0.0
ZD49	50.7***	6.0***	17.3***	20.8***	0.9***	3.5***	0.7***	0.03*
ZD59	50.5***	7.9***	18.5***	18.4***	0.8***	3.4***	0.6***	0.01
Phyll	4.0***	54.3***	15.5***	17.1***	1.3***	6.5***	1.3***	0.1
FLN	24.3***	57.7***	7.4***	8.2***	0.6***	1.6***	0.3***	0.0
PH	0.5*	9.5***	86.5***	0.2	1.5***	1.1***	0.8***	0.0
LIN	4.1***	47.6***	45.0***	0.4**	1.0***	1.4***	0.5***	0.0
SL	3.9***	18.8***	43.3***	15.2***	6.9***	6.0***	5.5***	0.5
RT	7.2***	77.0***	4.9***	7.2***	1.0***	1.9***	0.8***	0.1
ASN	6.9***	1.2**	38.7***	32.3***	4.8***	10.0***	6.0***	0.1
ASW	22.6***	27.9***	26.3***	13.8***	1.9***	4.5***	3.0***	0.0
ATKW	30.6***	49.7***	14.5***	2.2***	1.2***	1.0***	0.8***	0.0
GY	17.6***	45.0***	11.8***	20.4***	1.3***	3.0***	0.9***	0.0

[1] Trait abbreviations:

ZD31 – first node appearance
ZD37 – flag leaf emergence
ZD39 – flag leaf fully expanded
ZD49 – booting stage, ears in the flag leaf sheath
ZD59 – heading, the ears fully emerged from the flag leaf sheath
Phyll – speed of leafing out
FLN – final leaf number on the main stem
PH – final plant height of the main stem from soil surface to the bottom of the ear
LIN – last internode length on the main stem
SL – spike length on the main stem
RT – number of reproductive tillers
ASN – average grain number per spike
ASW – average grain weight per spike
ATKW – average thousand kernel weight
GY – total grain yield per plant

Supplementary Figure S1 Associations between the heading date values of the 19 wheat cultivars originating from two different sets of experiments referred to as DEV59 (heading) coming from the controlled growth chamber tests by Kiss et al. [12] or ZD59 coming from the present greenhouse experiment. (a) Comparisons between the constant 18 °C temperature regime of the growth chamber and the control greenhouse environment (DEV59_18C vs. ZD59_GH), (b) Comparisons between the constant 25 °C temperature regime of the growth chamber and the short-term 25 °C in fully vernalized seedling stage (v60) followed by greenhouse raising (DEV59_25C vs ZD59_25GH). ** indicates a significance level of P < 0.01, while *** indicates a significance level of P < 0.001.

(a)

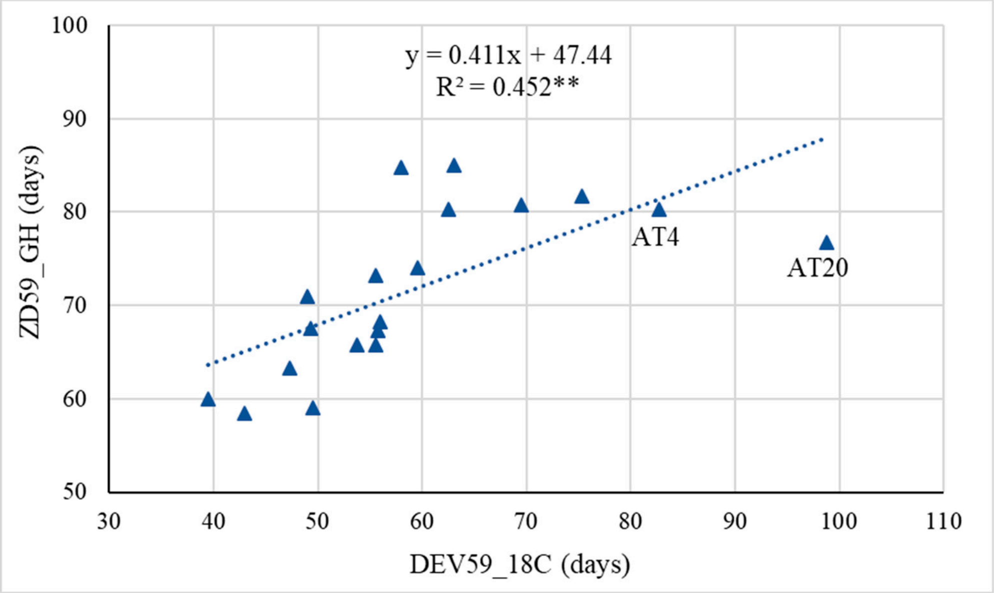

(b)

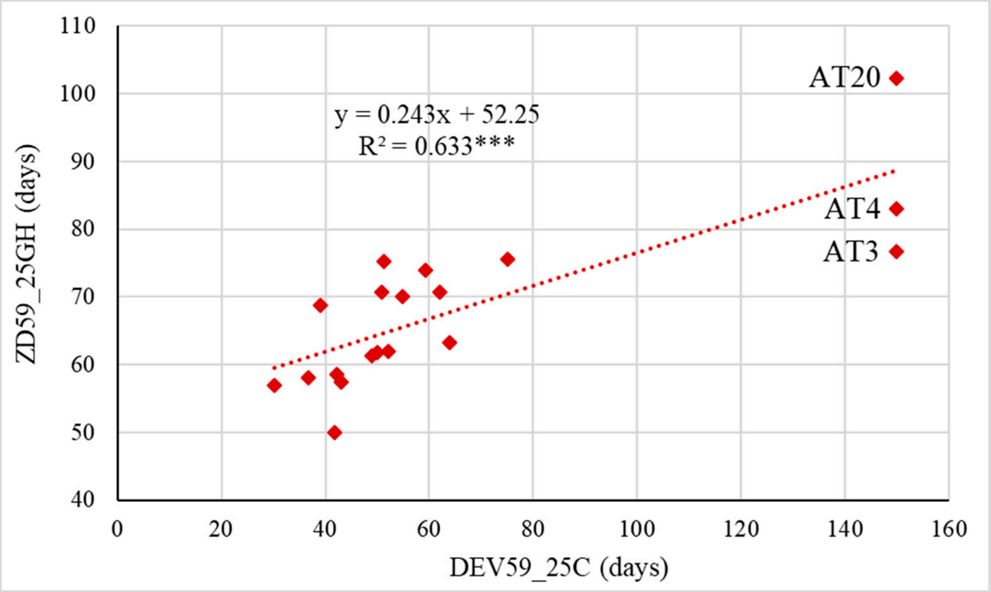

Supplementary Figure S2 Correlation matrices between the various developmental interval lengths and grain yield related traits in the group of 19 wheat cultivars under four environments combining two vernalization lengths (60- or 30-day) with two ambient temperature regimes (control greenhouse (GH) or two-week 25 °C followed by greenhouse conditions (25GH))

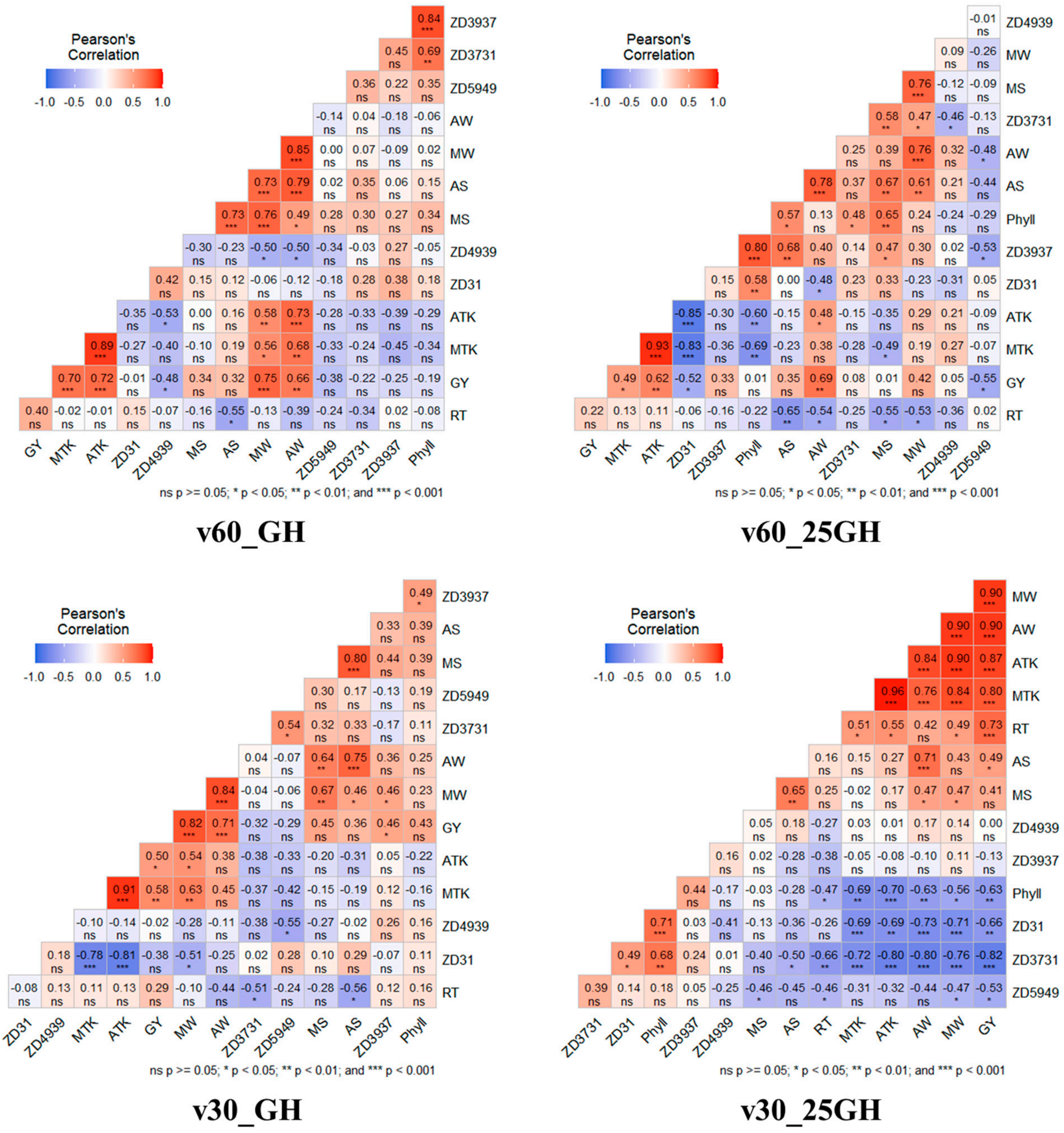

Trait abbreviations: ZD31 – appearance of the first main stem node, ZD3731 – the interval between appearance of the first main stem node and appearance of the flag leaf, ZD3937 – the interval between appearance of the flag leaf and full expansion of the flag leaf, ZD4939 – the interval between full expansion of the flag leaf and booting, ZD5949 – the interval between booting and heading, Phyll – phyllochron, RT – reproductive tiller number, MSN – number of grains on the main stem, MSW – weight of grains on the main stem, MTKW – thousand-kernel weight on the main stem, ASN – average seed number per spike, ASW – average seed weight per spike, ATKW – average thousand kernel weight, GY – plant grain yield

Supplementary Figure S3 Comparing the development of wheat plants grown under autumn-sown field conditions and in controlled growth chamber with floral inductive conditions (fully vernalized plants, 16-h photoperiod, 18 °C constant temperature). (a) correlation between the heading dates (ZD59) of 188 wheat genotypes forming the KALGEN panel under field conditions, averaged over three seasons between 2013-2015 [12,48] and in controlled experiment, (b) developmental characteristics of 'Mv Toborzó' (AT1) apices and plantlets after vernalization saturation under the two conditions. *** indicates a significance level of P < 0.001.

(a)

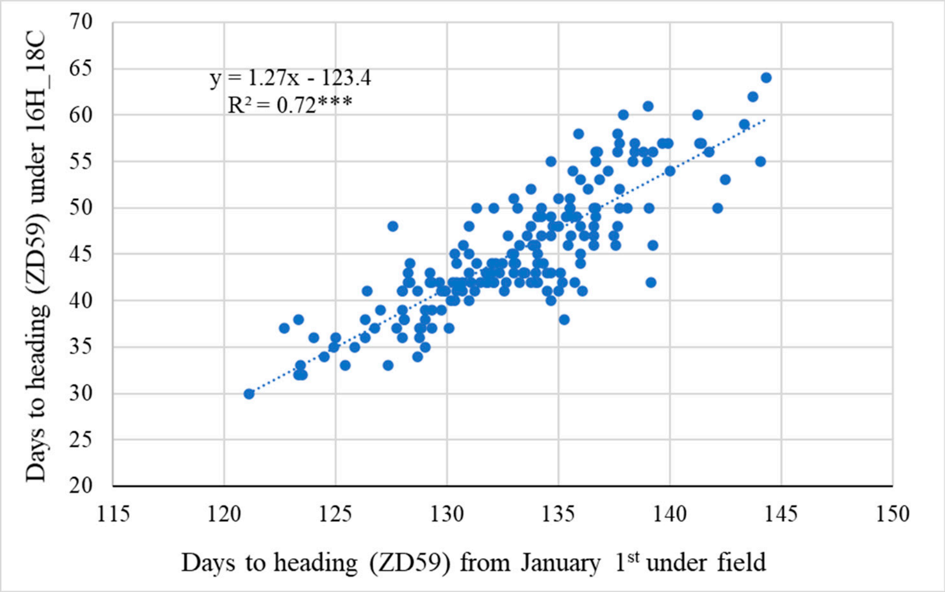

(b)

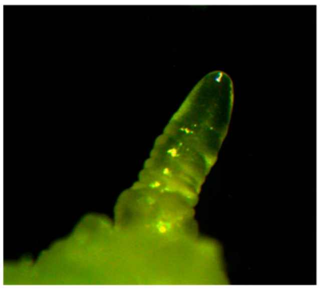

Field	Traits	Controlled
68 days from Jan 1.	**Sampling time**	After a 60-day vernalization
5.5	**No. of leaves visible**	2.0
3.5	**No. of inner leaves**	3.0
3.5	**No. of tillers**	0.0
6.0	**Plant height (cm)**	1.5
0.72	**Apex length (mm)**	0.37

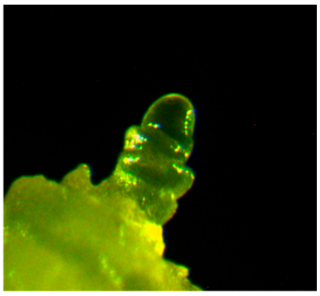

Supplement: Supplementary file 1 [file plants-15-02955-s001.zip › plants-4536812-supplementary.pdf]
